# Supplementary material for: Wide-Targeted Metabolome Analysis Identifies Potential Biomarkers for Prognosis Prediction of Epithelial Ovarian Cancer
Source: Toxins (Basel). 2021 Jun 30;13(7):461. doi: 10.3390/toxins13070461 (PMC8309959; doi:10.3390/toxins13070461)
Supplement: Supplementary file 1 [file toxins-13-00461-s001.zip › toxins-1264119-supplementary.pdf]

Supplementary Materials: Wide-Targeted Metabolome Analysis Identifies Potential Biomarkers for Prognosis Prediction of Epithelial Ovarian Cancer

Eiji Hishinuma, Muneaki Shimada, Naomi Matsukawa, Daisuke Saigusa, Bin Li, Kei Kudo, Keita Tsuji, Shogo Shigeta, Hideki Tokunaga, Kazuki Kumada, Keigo Komine, Hidekazu Shirota, Yuichi Aoki, Ikuko N. Motoike, Jun Yasuda, Kengo Kinoshita, Masayuki Yamamoto, Seizo Koshiba and Nobuo Yaegashi

Table S1. Significantly increased metabolites in the plasma of epithelial ovarian cancer patients.

Metabolite	Class	Cancer (µM, mean ± SD)	Cohort (µM, mean ± SD)	p-value	Fold change (Cancer/Cohort)
TG (16:0_40:6)	Triglycerides	2.72 ± 1.51	1.05 ± 0.45	1.43×10^{-16}	2.58
Hypoxanthine	Nucleobases and related	9.43 ± 22.55	3.88 ± 2.17	4.44×10^{-2}	2.43
Cortisol	Hormones and related	0.31 ± 0.10	0.16 ± 0.06	4.35×10^{-24}	1.94
TG (18:1_36:2)	Triglycerides	64.19 ± 33.17	36.42 ± 22.01	3.89×10^{-9}	1.76
TG (22:5_34:2)	Triglycerides	1.94 ± 0.85	1.11 ± 0.63	1.40×10^{-11}	1.75
TG (18:2_36:2)	Triglycerides	29.32 ± 15.10	17.18 ± 11.01	3.33×10^{-8}	1.71
TG (18:1_34:1)	Triglycerides	193.84 ± 98.13	114.61 ± 71.77	3.04×10^{-8}	1.69
TG (18:1_36:3)	Triglycerides	52.68 ± 27.98	31.15 ± 20.70	1.29×10^{-7}	1.69
TG (20:4_34:1)	Triglycerides	5.49 ± 2.33	3.31 ± 2.16	3.19×10^{-9}	1.66
TG (16:0_36:2)	Triglycerides	99.75 ± 50.83	60.19 ± 38.32	1.13×10^{-7}	1.66
TG (16:0_36:3)	Triglycerides	99.69 ± 48.48	60.21 ± 37.58	4.34×10^{-8}	1.66
TG (18:2_34:1)	Triglycerides	99.06 ± 47.69	59.91 ± 36.66	3.15×10^{-8}	1.65
GLCAS	Bile acids	0.27 ± 0.27	0.16 ± 0.14	2.60×10^{-3}	1.65
TG (18:0_36:3)	Triglycerides	9.82 ± 4.43	5.98 ± 3.64	9.19×10^{-9}	1.64
TG (18:1_34:2)	Triglycerides	110.15 ± 52.06	67.77 ± 40.23	4.23×10^{-8}	1.63
TG (18:0_36:2)	Triglycerides	8.60 ± 3.94	5.30 ± 3.53	4.79×10^{-8}	1.62
SM C26:1	Sphingomyelines	1.74 ± 0.81	1.08 ± 0.33	1.78×10^{-10}	1.62
TG (18:2_36:1)	Triglycerides	10.62 ± 4.89	6.65 ± 4.10	7.12×10^{-8}	1.60
TG (18:1_36:1)	Triglycerides	17.47 ± 8.41	10.97 ± 7.18	3.18×10^{-7}	1.59
TG (18:2_35:1)	Triglycerides	1.64 ± 0.79	1.07 ± 0.50	1.95×10^{-8}	1.53

TG (18:2_34:2)	Triglycerides	50.08 ± 25.05	32.81 ± 21.08	5.24×10^{-6}	1.53
TG (18:2_34:0)	Triglycerides	9.98 ± 4.60	6.65 ± 4.74	9.41×10^{-6}	1.50
TG (22:5_34:1)	Triglycerides	4.30 ± 2.00	2.88 ± 1.28	3.10×10^{-9}	1.49
TG (16:1_36:2)	Triglycerides	13.15 ± 6.87	8.83 ± 5.25	1.09×10^{-5}	1.49
TG (18:2_36:3)	Triglycerides	19.71 ± 11.82	13.29 ± 10.31	3.42×10^{-4}	1.48
TG (18:1_35:2)	Triglycerides	3.20 ± 1.57	2.21 ± 0.97	7.02×10^{-7}	1.45
TG (16:0_36:4)	Triglycerides	28.20 ± 14.55	19.99 ± 14.03	3.74×10^{-4}	1.41
TG (17:2_38:6)	Triglycerides	0.35 ± 0.17	0.25 ± 0.13	5.18×10^{-5}	1.39
TG (17:2_38:5)	Triglycerides	0.37 ± 0.16	0.26 ± 0.14	1.09×10^{-4}	1.39
TG (22:6_32:1)	Triglycerides	1.78 ± 1.06	1.32 ± 1.53	7.41×10^{-3}	1.35
Kynurenine	Amino acid related	2.04 ± 0.87	1.52 ± 0.57	1.25×10^{-5}	1.35
TG (18:1_33:1)	Triglycerides	3.74 ± 1.93	2.80 ± 1.35	1.23×10^{-4}	1.34
HCys	Amino acid related	27.69 ± 17.04	20.80 ± 12.81	4.37×10^{-3}	1.33
C18:1	Acylcarnitines	0.11 ± 0.05	0.09 ± 0.03	9.05×10^{-3}	1.32
TG (18:2_33:1)	Triglycerides	1.95 ± 0.82	1.47 ± 0.65	4.51×10^{-6}	1.32
TG (17:0_36:3)	Triglycerides	1.51 ± 0.69	1.15 ± 0.51	9.40×10^{-5}	1.32
TG (18:1_32:1)	Triglycerides	32.05 ± 16.03	24.39 ± 17.21	4.11×10^{-3}	1.31
TG (18:1_36:4)	Triglycerides	15.92 ± 9.03	12.18 ± 8.52	7.91×10^{-3}	1.31
TG (16:1_34:1)	Triglycerides	17.85 ± 8.15	13.70 ± 10.25	1.13×10^{-2}	1.30
TG (20:4_34:2)	Triglycerides	2.89 ± 1.12	2.22 ± 1.55	4.54×10^{-4}	1.30
TG (18:0_36:4)	Triglycerides	3.79 ± 1.68	2.91 ± 1.62	7.98×10^{-4}	1.30
TG (16:0_34:2)	Triglycerides	36.37 ± 17.48	28.27 ± 23.18	1.36×10^{-2}	1.29
TG (16:0_40:7)	Triglycerides	1.80 ± 1.47	1.40 ± 0.95	1.19×10^{-2}	1.29
t4-OH-Pro	Amino acid related	9.99 ± 4.72	7.77 ± 2.41	2.50×10^{-4}	1.29
Cer (d18:1/24:1)	Ceramides	2.13 ± 0.56	1.66 ± 0.34	1.11×10^{-9}	1.29
Hex2Cer (d18:1/24:1)	Dihexosylceramides	0.51 ± 0.17	0.40 ± 0.11	3.00×10^{-5}	1.28
TG (20:4_36:4)	Triglycerides	0.69 ± 0.27	0.54 ± 0.29	1.31×10^{-3}	1.28
TG (16:1_36:1)	Triglycerides	2.47 ± 1.03	1.94 ± 1.05	1.24×10^{-4}	1.28
TG (20:2_34:1)	Triglycerides	1.33 ± 0.54	1.05 ± 0.54	1.04×10^{-4}	1.27
DG (18:2_18:2)	Diglycerides	3.90 ± 1.78	3.06 ± 1.47	5.72×10^{-4}	1.27
TG (18:2_38:5)	Triglycerides	1.34 ± 0.53	1.06 ± 0.39	1.77×10^{-4}	1.27

TG (20:4_36:2)	Triglycerides	2.52 ± 0.98	1.99 ± 0.86	1.26×10^{-4}	1.26
TG (16:1_36:3)	Triglycerides	11.34 ± 5.51	9.01 ± 4.74	5.17×10^{-4}	1.26
beta-Ala	Biogenic amines	2.03 ± 1.13	1.63 ± 1.15	2.58×10^{-2}	1.25
TG (18:1_32:0)	Triglycerides	25.61 ± 12.88	20.53 ± 19.35	3.96×10^{-2}	1.25
TG (20:5_34:1)	Triglycerides	2.15 ± 1.01	1.72 ± 1.36	1.02×10^{-2}	1.25
TG (18:0_34:2)	Triglycerides	6.28 ± 2.57	5.06 ± 3.78	1.09×10^{-2}	1.24
TG (18:1_35:3)	Triglycerides	0.92 ± 0.49	0.74 ± 0.29	1.88×10^{-3}	1.24
TG (17:2_36:4)	Triglycerides	0.71 ± 0.35	0.58 ± 0.34	7.78×10^{-3}	1.24
Thr	Amino acids	138.81 ± 45.28	112.52 ± 24.63	1.02×10^{-5}	1.23
CE (22:5)	Cholesteryl esters	2.88 ± 1.79	2.34 ± 1.14	1.13×10^{-2}	1.23
TG (16:1_34:2)	Triglycerides	10.41 ± 4.35	8.49 ± 5.77	3.12×10^{-2}	1.23
TG (18:3_34:3)	Triglycerides	0.97 ± 0.52	0.80 ± 0.47	2.24×10^{-2}	1.22
TG (18:2_32:1)	Triglycerides	13.91 ± 6.42	11.54 ± 7.68	3.61×10^{-2}	1.20
TG (18:2_36:0)	Triglycerides	1.01 ± 0.43	0.86 ± 0.48	3.58×10^{-3}	1.17
SDMA	Amino acid related	0.54 ± 0.17	0.46 ± 0.08	2.86×10^{-4}	1.17
Asp	Amino acids	7.19 ± 4.39	6.15 ± 1.22	4.16×10^{-2}	1.17
ADMA	Amino acid related	0.52 ± 0.10	0.45 ± 0.13	1.67×10^{-4}	1.16
Ile	Amino acids	73.33 ± 20.92	64.53 ± 18.19	5.08×10^{-3}	1.14
SM C18:0	Sphingomyelines	41.84 ± 10.99	37.00 ± 8.32	2.01×10^{-3}	1.13
TG (18:3_34:0)	Triglycerides	1.11 ± 0.53	0.98 ± 0.62	2.02×10^{-2}	1.13
Gly	Amino acids	271.08 ± 82.02	240.34 ± 78.47	1.66×10^{-2}	1.13
C16:1	Acylcarnitines	0.04 ± 0.02	0.03 ± 0.01	2.41×10^{-3}	1.13
TG (16:0_38:2)	Triglycerides	1.35 ± 0.78	1.21 ± 0.81	3.87×10^{-2}	1.11
TG (20:4_32:0)	Triglycerides	0.92 ± 0.43	0.83 ± 0.63	3.22×10^{-2}	1.11
TG (16:0_38:3)	Triglycerides	1.55 ± 0.78	1.40 ± 0.83	4.42×10^{-2}	1.11
Hex2Cer(d18:1/16:0)	Dihexosylceramides	1.99 ± 0.56	1.80 ± 0.39	1.12×10^{-2}	1.11
Lys	Amino acids	186.06 ± 46.15	169.93 ± 47.05	3.01×10^{-2}	1.09
Cer(d18:1/25:0)	Ceramides	0.37 ± 0.09	0.34 ± 0.10	2.73×10^{-2}	1.09

Table S2. Significantly decreased metabolites in the plasma of epithelial ovarian cancer patients.

Metabolite	Class	Cancer (μM, mean ± SD)	Cohort (μM, mean ± SD)	*p*-value	Fold change (Cancer/Cohort)
PC ae C38:2	Phosphatidylcholines	2.39 ± 2.14	7.00 ± 1.29	6.15×10^{-37}	0.34
Serotonin	Biogenic amines	0.12 ± 0.08	0.30 ± 0.20	8.01×10^{-12}	0.40
CE (20:5)	Cholesteryl esters	61.64 ± 39.16	144.24 ± 106.50	9.43×10^{-10}	0.43
DHA	Fatty acids	12.03 ± 16.07	26.67 ± 34.72	7.86×10^{-4}	0.45
PC ae C38:3	Phosphatidylcholines	4.35 ± 1.93	9.54 ± 1.84	1.80×10^{-38}	0.46
PC aa C40:2	Phosphatidylcholines	0.24 ± 0.16	0.50 ± 0.13	4.76×10^{-22}	0.47
PC ae C40:3	Phosphatidylcholines	1.55 ± 0.75	3.07 ± 0.72	8.18×10^{-27}	0.50
PC ae C38:1	Phosphatidylcholines	1.22 ± 1.19	2.34 ± 1.03	1.52×10^{-9}	0.52
AA	Fatty acids	1.76 ± 1.20	3.29 ± 2.32	1.10×10^{-6}	0.54
Cer(d18:2/23:0)	Ceramides	0.12 ± 0.06	0.21 ± 0.13	1.96×10^{-8}	0.55
PC aa C42:4	Phosphatidylcholines	0.15 ± 0.05	0.26 ± 0.13	8.63×10^{-12}	0.56
TG (22:6_34:3)	Triglycerides	0.82 ± 0.74	1.42 ± 0.95	1.80×10^{-3}	0.58
PC aa C34:4	Phosphatidylcholines	0.50 ± 0.26	0.84 ± 0.24	1.02×10^{-14}	0.60
CE (18:3)	Cholesteryl esters	37.41 ± 18.70	60.12 ± 22.13	6.58×10^{-11}	0.62
PC ae C40:1	Phosphatidylcholines	0.68 ± 0.29	1.09 ± 0.26	7.94×10^{-17}	0.62
PC aa C42:2	Phosphatidylcholines	0.23 ± 0.09	0.36 ± 0.10	1.43×10^{-16}	0.62
C14:1	Acylcarnitines	0.04 ± 0.02	0.06 ± 0.02	2.12×10^{-11}	0.64
PC aa C40:3	Phosphatidylcholines	0.59 ± 0.26	0.92 ± 0.25	8.64×10^{-14}	0.64
PC ae C44:3	Phosphatidylcholines	0.08 ± 0.03	0.13 ± 0.04	6.63×10^{-15}	0.65
TrpBetaine	Amino acid related	0.11 ± 0.14	0.17 ± 0.18	2.60×10^{-2}	0.65
PC aa C36:0	Phosphatidylcholines	2.10 ± 1.02	3.22 ± 1.29	6.90×10^{-9}	0.65
lysoPC a C20:3	Lysophosphatidylcholines	0.87 ± 0.32	1.31 ± 0.45	1.64×10^{-11}	0.66
PC ae C40:4	Phosphatidylcholines	1.27 ± 0.43	1.93 ± 0.42	4.96×10^{-18}	0.66
lysoPC a C24:0	Lysophosphatidylcholines	0.15 ± 0.04	0.22 ± 0.06	7.78×10^{-15}	0.67
PC aa C36:6	Phosphatidylcholines	0.49 ± 0.27	0.74 ± 0.29	1.37×10^{-7}	0.67
PC ae C42:1	Phosphatidylcholines	0.22 ± 0.06	0.32 ± 0.06	3.61×10^{-21}	0.67
PC ae C30:2	Phosphatidylcholines	0.45 ± 0.20	0.67 ± 0.14	7.98×10^{-13}	0.67

lysoPC a C18:2	Lysophosphatidylcholines	11.76 ± 5.74	17.49 ± 6.26	1.13×10^{-8}	0.67
p-Cresol-SO4	Cresols	14.18 ± 10.97	21.05 ± 17.34	3.44×10^{-3}	0.67
lysoPC a C18:0	Lysophosphatidylcholines	19.86 ± 8.02	29.47 ± 7.54	7.28×10^{-13}	0.67
PC ae C42:3	Phosphatidylcholines	0.61 ± 0.21	0.90 ± 0.19	1.25×10^{-16}	0.67
DHEAS	Hormones and related	1.57 ± 1.01	2.32 ± 1.34	8.55×10^{-5}	0.67
PC ae C42:4	Phosphatidylcholines	0.48 ± 0.23	0.71 ± 0.19	7.03×10^{-11}	0.68
PC aa C32:2	Phosphatidylcholines	3.38 ± 1.79	4.94 ± 0.95	1.28×10^{-10}	0.68
PC aa C42:5	Phosphatidylcholines	0.30 ± 0.12	0.44 ± 0.12	1.71×10^{-11}	0.69
Cer(d18:2/24:0)	Ceramides	0.42 ± 0.16	0.60 ± 0.18	1.53×10^{-10}	0.70
lysoPC a C16:1	Lysophosphatidylcholines	0.98 ± 0.40	1.40 ± 0.42	8.81×10^{-10}	0.70
PC ae C34:3	Phosphatidylcholines	4.08 ± 1.57	5.77 ± 1.55	1.55×10^{-10}	0.71
PC ae C42:2	Phosphatidylcholines	0.40 ± 0.15	0.56 ± 0.11	1.49×10^{-11}	0.72
Cit	Amino acid related	24.21 ± 8.32	33.46 ± 10.69	7.45×10^{-9}	0.72
lysoPC a C28:0	Lysophosphatidylcholines	0.15 ± 0.04	0.21 ± 0.08	2.09×10^{-7}	0.73
PC aa C40:4	Phosphatidylcholines	2.01 ± 0.56	2.76 ± 0.70	5.80×10^{-12}	0.73
PC ae C34:2	Phosphatidylcholines	6.19 ± 1.83	8.44 ± 2.05	1.25×10^{-11}	0.73
PC ae C36:1	Phosphatidylcholines	11.95 ± 3.27	16.28 ± 3.19	1.54×10^{-14}	0.73
lysoPC a C17:0	Lysophosphatidylcholines	1.06 ± 0.49	1.45 ± 0.38	9.64×10^{-8}	0.73
PC aa C42:1	Phosphatidylcholines	0.25 ± 0.09	0.34 ± 0.09	5.09×10^{-9}	0.74
PC ae C38:0	Phosphatidylcholines	1.17 ± 0.47	1.58 ± 0.43	2.98×10^{-8}	0.74
PC ae C40:5	Phosphatidylcholines	2.69 ± 0.77	3.64 ± 0.90	2.43×10^{-11}	0.74
TG (17:0_34:1)	Triglycerides	0.97 ± 0.33	1.31 ± 0.63	1.98×10^{-4}	0.74
PC ae C36:3	Phosphatidylcholines	3.75 ± 1.13	5.02 ± 1.11	2.63×10^{-11}	0.75
SM C24:0	Sphingomyelines	23.60 ± 5.43	31.41 ± 6.45	4.81×10^{-14}	0.75
PC aa C32:3	Phosphatidylcholines	0.74 ± 0.24	0.98 ± 0.20	1.94×10^{-10}	0.76
PC ae C38:6	Phosphatidylcholines	4.45 ± 1.56	5.87 ± 1.51	2.75×10^{-8}	0.76
lysoPC a C26:1	Lysophosphatidylcholines	0.09 ± 0.03	0.12 ± 0.04	9.38×10^{-7}	0.76
TG (20:3_36:4)	Triglycerides	0.28 ± 0.14	0.37 ± 0.22	1.36×10^{-3}	0.76
lysoPC a C18:1	Lysophosphatidylcholines	11.75 ± 4.55	15.37 ± 4.20	5.48×10^{-7}	0.76
SM C26:0	Sphingomyelines	0.56 ± 0.16	0.73 ± 0.19	3.13×10^{-9}	0.76
lysoPC a C16:0	Lysophosphatidylcholines	45.75 ± 16.73	59.39 ± 14.31	1.22×10^{-7}	0.77

Cer(d18:2/22:0)	Ceramides	0.32 ± 0.12	0.41 ± 0.15	2.26×10^{-5}	0.77
PC ae C36:5	Phosphatidylcholines	6.17 ± 2.05	7.97 ± 1.95	5.40×10^{-8}	0.77
PC aa C38:3	Phosphatidylcholines	36.30 ± 9.40	46.75 ± 9.53	7.56×10^{-11}	0.78
lysoPC a C28:1	Lysophosphatidylcholines	0.21 ± 0.07	0.26 ± 0.10	2.74×10^{-5}	0.78
PC ae C36:4	Phosphatidylcholines	7.97 ± 2.32	10.20 ± 2.42	1.74×10^{-8}	0.78
CE (22:6)	Cholesteryl esters	46.44 ± 23.63	59.33 ± 36.59	9.93×10^{-3}	0.78
His	Amino acids	64.02 ± 16.53	81.59 ± 11.26	5.61×10^{-13}	0.78
lysoPC a C26:0	Lysophosphatidylcholines	0.17 ± 0.05	0.22 ± 0.08	2.02×10^{-5}	0.79
PC aa C36:5	Phosphatidylcholines	20.93 ± 10.56	26.51 ± 12.79	3.08×10^{-3}	0.79
PC aa C38:5	Phosphatidylcholines	33.69 ± 9.66	42.66 ± 11.82	4.74×10^{-7}	0.79
PC aa C40:6	Phosphatidylcholines	34.44 ± 10.45	43.45 ± 13.17	3.77×10^{-6}	0.79
TG (18:1_38:7)	Triglycerides	0.28 ± 0.15	0.35 ± 0.11	7.29×10^{-4}	0.79
SM (OH) C22:2	Sphingomyelines	12.18 ± 2.95	15.30 ± 3.54	9.42×10^{-9}	0.80
C2	Acylcarnitines	5.67 ± 2.92	7.13 ± 2.72	1.76×10^{-3}	0.80
SM (OH) C24:1	Sphingomyelines	2.56 ± 0.72	3.21 ± 0.73	6.37×10^{-8}	0.80
lysoPC a C20:4	Lysophosphatidylcholines	2.80 ± 1.11	3.51 ± 0.84	1.14×10^{-5}	0.80
PC aa C34:3	Phosphatidylcholines	5.34 ± 2.09	6.69 ± 1.66	1.22×10^{-5}	0.80
PC aa C42:0	Phosphatidylcholines	0.54 ± 0.21	0.68 ± 0.18	3.22×10^{-5}	0.80
Taurine	Amino acid related	75.51 ± 34.51	94.00 ± 24.49	1.38×10^{-4}	0.80
Cer (d18:1/23:0)	Ceramides	0.82 ± 0.22	1.01 ± 0.26	6.28×10^{-7}	0.81
PC ae C40:2	Phosphatidylcholines	2.11 ± 0.66	2.62 ± 0.64	2.00×10^{-6}	0.81
PC ae C30:0	Phosphatidylcholines	0.22 ± 0.07	0.27 ± 0.08	1.59×10^{-5}	0.81
PC aa C36:1	Phosphatidylcholines	34.39 ± 8.83	42.49 ± 10.48	4.09×10^{-7}	0.81
PC aa C38:0	Phosphatidylcholines	3.02 ± 1.01	3.72 ± 1.05	3.28×10^{-5}	0.81
CE (18:0)	Cholesteryl esters	8.46 ± 4.19	10.40 ± 3.51	3.21×10^{-3}	0.81
PC aa C28:1	Phosphatidylcholines	2.60 ± 0.71	3.18 ± 0.69	4.20×10^{-7}	0.82
CE (20:3)	Cholesteryl esters	14.33 ± 4.25	17.52 ± 5.05	1.11×10^{-4}	0.82
PC aa C40:5	Phosphatidylcholines	6.78 ± 1.98	8.28 ± 2.41	3.06×10^{-5}	0.82
SM (OH) C22:1	Sphingomyelines	12.63 ± 3.10	15.37 ± 3.35	2.92×10^{-7}	0.82
CE (18:1)	Cholesteryl esters	208.33 ± 64.50	253.04 ± 65.21	2.34×10^{-5}	0.82
Val	Amino acids	166.82 ± 46.80	201.59 ± 40.01	1.20×10^{-6}	0.83

PC ae C40:6	Phosphatidylcholines	3.99 ± 1.30	4.80 ± 1.17	5.63×10^{-5}	0.83
PC aa C36:3	Phosphatidylcholines	67.87 ± 18.73	81.50 ± 18.98	9.72×10^{-6}	0.83
PC aa C38:6	Phosphatidylcholines	90.50 ± 27.54	108.56 ± 27.44	5.30×10^{-5}	0.83
Trp	Amino acids	43.51 ± 11.89	52.17 ± 9.22	7.85×10^{-7}	0.83
PC aa C30:0	Phosphatidylcholines	3.27 ± 1.11	3.92 ± 1.02	1.74×10^{-4}	0.83
PC ae C36:2	Phosphatidylcholines	8.86 ± 2.32	10.59 ± 2.31	4.82×10^{-6}	0.84
Cer (d18:1/24:0)	Ceramides	2.10 ± 0.58	2.50 ± 0.63	3.74×10^{-5}	0.84
PC aa C36:2	Phosphatidylcholines	168.44 ± 40.84	200.78 ± 40.91	1.49×10^{-6}	0.84
PC ae C36:0	Phosphatidylcholines	0.62 ± 0.18	0.74 ± 0.17	2.58×10^{-5}	0.84
DG (17:0_18:1)	Diglycerides	0.45 ± 0.16	0.53 ± 0.17	3.12×10^{-3}	0.84
HArg	Amino acid related	5.30 ± 2.91	6.26 ± 1.89	1.48×10^{-2}	0.85
PC aa C32:1	Phosphatidylcholines	9.44 ± 3.77	11.13 ± 3.96	6.42×10^{-3}	0.85
TG (18:0_36:5)	Triglycerides	0.73 ± 0.26	0.86 ± 0.39	2.48×10^{-2}	0.86
PC aa C38:4	Phosphatidylcholines	65.39 ± 16.86	75.51 ± 16.59	1.87×10^{-4}	0.87
PC ae C44:4	Phosphatidylcholines	0.26 ± 0.10	0.30 ± 0.06	3.00×10^{-3}	0.87
PC ae C38:4	Phosphatidylcholines	6.22 ± 1.71	7.19 ± 1.38	1.32×10^{-4}	0.87
PC ae C42:5	Phosphatidylcholines	1.55 ± 0.46	1.77 ± 0.38	1.81×10^{-4}	0.87
PC ae C32:1	Phosphatidylcholines	1.66 ± 0.44	1.88 ± 0.39	1.16×10^{-3}	0.88
CE (20:4)	Cholesteryl esters	139.76 ± 52.16	157.98 ± 51.80	2.80×10^{-2}	0.88
CE (16:0)	Cholesteryl esters	131.34 ± 37.06	148.25 ± 35.19	3.56×10^{-3}	0.89
PC ae C38:5	Phosphatidylcholines	9.23 ± 2.33	10.37 ± 2.00	1.12×10^{-3}	0.89
PC ae C32:2	Phosphatidylcholines	0.89 ± 0.24	0.99 ± 0.22	4.22×10^{-3}	0.89
Tyr	Amino acids	52.74 ± 14.27	58.63 ± 12.62	6.41×10^{-3}	0.90
PC ae C44:6	Phosphatidylcholines	0.88 ± 0.33	0.97 ± 0.23	3.17×10^{-2}	0.90
Gln	Amino acids	648.70 ± 124.61	689.46 ± 94.32	2.09×10^{-2}	0.94
